# Chemical Chaperones Improve Protein Secretion and Rescue Mutant Factor VIII in Mice with Hemophilia A

**DOI:** 10.1371/journal.pone.0044505

**Published:** 2012-09-04

**Authors:** Stefanie D. Roth, Jörg Schüttrumpf, Peter Milanov, Daniela Abriss, Christopher Ungerer, Patricia Quade-Lyssy, Jeremy C. Simpson, Rainer Pepperkok, Erhard Seifried, Torsten Tonn

**Affiliations:** 1 Institute for Transfusion Medicine and Immune Hematology, Clinics of the Johann Wolfgang Goethe University, German Red Cross Blood Donor Service Baden-Wuerttemberg - Hessen, Frankfurt am Main, Hesse, Germany; 2 Biomedical Research Institute Georg-Speyer-Haus, Frankfurt am Main, Hesse, Germany; 3 School of Biology and Environmental Science, University College Dublin, Dublin, Ireland; 4 Cell Biology and Biophysics Department, European Molecular Biology Laboratory (EMBL), Heidelberg, Baden-Wuerttemberg, Germany; Emory University School of Medicine, United States of America

## Abstract

Inefficient intracellular protein trafficking is a critical issue in the pathogenesis of a variety of diseases and in recombinant protein production. Here we investigated the trafficking of factor VIII (FVIII), which is affected in the coagulation disorder hemophilia A. We hypothesized that chemical chaperones may be useful to enhance folding and processing of FVIII in recombinant protein production, and as a therapeutic approach in patients with impaired FVIII secretion. A tagged B-domain-deleted version of human FVIII was expressed in cultured Chinese Hamster Ovary cells to mimic the industrial production of this important protein. Of several chemical chaperones tested, the addition of betaine resulted in increased secretion of FVIII, by increasing solubility of intracellular FVIII aggregates and improving transport from endoplasmic reticulum to Golgi. Similar results were obtained in experiments monitoring recombinant full-length FVIII. Oral betaine administration also increased FVIII and factor IX (FIX) plasma levels in FVIII or FIX knockout mice following gene transfer. Moreover, *in vitro* and *in vivo* applications of betaine were also able to rescue a trafficking-defective FVIII mutant (FVIIIQ305P). We conclude that chemical chaperones such as betaine might represent a useful treatment concept for hemophilia and other diseases caused by deficient intracellular protein trafficking.

## Introduction

Low molecular weight compounds termed “chemical chaperones” (CC) can inhibit aggregation and restore trafficking of intracellularly misfolded proteins. They stabilize native protein conformations non-specifically and support escape from endoplasmic quality control systems [1], [2]. CC are currently under investigation for treatment of certain proteopathies or so called conformational disorders [3] such as nephrogenic diabetes insipidus caused by vasopressin V2 receptor mutants [4] or diabetes mellitus [5]. The CC sodium-4-phenylbutyrate has already been approved for phase II clinical trials for the treatment of cystic fibrosis [6], [7]. Betaine is under clinical evaluation for treatment of primary hyperoxaluria [8].

In hemophilia A, protein substitution with recombinant factor VIII (rFVIII) concentrate is the treatment of choice. It is estimated that due to high treatment costs, only 30% of affected individuals worldwide receive any kind of hemophilia therapy [9]. This fact highlights the necessity for alternative strategies that can improve the clinical outcome, reduce factor consumption or decrease production costs. rFVIII cannot be produced in bacterial systems, because diverse post-translational modifications and complex formation are necessary for FVIII functionality [10]. Therefore, industrial production relies on mammalian cells that provide the essential enzyme repertoire for mature FVIII-heterodimer synthesis. In such cell factories, secretion of recombinant FVIII remains inefficient, as a result of several limiting factors. These include low FVIII mRNA-levels (with heterologous expression levels 2 to 3 orders of magnitude below other recombinant proteins [11], [12], inefficient translation of the large FVIII transcript, and poor transport rates between the endoplasmic reticulum (ER) and the Golgi apparatus [12], [13]. We have previously observed in confocal microscopy studies, that the bulk of the translated protein is retained in ER or ERGIC [13]. The ER has a limited capacity to handle protein load, and overexpression of transgenes leads to partially folded polypeptides. Within the ER of cells, a quality control system employing molecular chaperones has the task of preventing transit of such mis-folded proteins from ER to Golgi [14]. Swaroop et al. have shown that FVIII interacts with the chaperones immunoglobulin-binding protein (BiP/GRP78) [15], calnexin, and calreticulin [16], which results in formation of non-disulfide-bonded high molecular weight aggregates in the ER. These misfolded FVIII proteins are subsequently designated for proteasomal and lysosomal degradation [17]. Nevertheless, a number of different modifications in recombinant FVIII have helped increase secretion efficiency. After the large B domain structure was identified as dispensable for FVIII coagulant activity, B-domain deletion (BDD) has been used as a strategy for improved intracellular trafficking of rhFVIII. Despite 20-fold higher mRNA expression levels, the additional yield of secreted B-domain deleted FVIII was only increased to between 30 and 50% [18]. Reintroduction of 226 amino acids of the B-domain with 6 N-linked oligosaccharides, and mutation of the BiP binding site at Phe309, further enhanced FVIII secretion [15], [19], confirming the critical role of the glycosylation status and chaperone interaction for FVIII folding and trafficking. Similar limitations have been observed in FVIII gene transfer approaches [20], [21].

We hypothesized that CC would increase the efficacy of mature rFVIII protein production and might benefit the treatment of hemophilia A caused by secretion-defective FVIII mutations. Testing various CC on their effect on FVIII secretion, the amino acid derivative betaine emerged as the strongest modifier of secretion levels. Since betaine is not cytotoxic, frequently used as food supplement, and already used in patients suffering from hyperhomocysteinemia, we further investigated oral administration of betaine in murine models of hemophilia A and B. In this context trafficking-defective FVIII were investigated. Missense mutations in all domains of FVIII can impair folding of FVIII and disturb its intracellular transport, which results in reduced FVIII plasma levels. Hemophilia A patients with missense mutations present with clinical phenotypes ranging from mild to severe [22]. Often they are characterized by low circulating antigen levels while many mutated proteins retain functionality. Here, rescue of trafficking-defective FVIII mutants was documented *in vitro* as well as *in vivo*. Prophylactic treatment studies have demonstrated that factor levels above one percent are sufficient to prevent most of the long term implications of hemophilia, such as disabling arthropathy and muscular atrophy [23]. Thus even a minimal increment of FVIII or FIX levels by alternative treatment strategies, such as proposed here, could provide relevant clinical benefit.

## Materials and Methods

### Ethics Statement

All animal studies were carried out in strict accordance with the recommendations in the Guide for the Care and Use of Laboratory Animals of the National Institutes of Health. All animal procedures were approved by the animal protection and use authority Regierungspraesidium Darmstadt (Approval number F27/08).

### Animal Experiments

FVIII-deficient mice with an exon 16 disruption on a C56Bl/6 background [24] were ordered by Charles River GmbH (Sulzfeld, Germany). FIX-deficient mice on a C57Bl/6 background were kindly provided by Katherine High [25]. Minicircle DNA was injected hydrodynamically via the tail vein as previously described [26] and 2% (w/v) anhydrous betaine was administered solubilized in drinking water ad libitum for defined time periods: Drinking water was replenished at least every 48 h. FVIII knockout mice received hydrodynamic injections of 20 µg of Minicircle-DNA expressing hFVIII-BDD or FVIII-BDDQ305P. Using a cross-over design, 24 hours following gene transfer, mice were randomly assigned to the control or the betaine-treated group for 3 days, then plasma samples were retroorbitally collected and treatment was switched between the groups for another 3 day-interval. In FIX knockout mice 17.6 µg (50 pmol) of pSL-hFIX vector were administered. Treatment occurred for 3 and then further 14 days until sampling, followed by a one-week interruption with subsequent crossover of groups for another 3 plus 14 days.

### Cell Lines and Culture Conditions

Chinese Hamster Ovary (ACC 110) and HepG2 (ACC 180) cell lines were maintained in Dulbecco’s modified Eagle medium supplemented with 2 mM L-glutamine, 100 IU/ml penicillin, 100 µg/ml streptomycin, and between 10 and 20% heat-inactivated fetal bovine serum (FBS) (Invitrogen, Belgium).

### Chemical Chaperones

Betaine (B14290), butylated hydroxyanisole (B1253), curcumin (C7727) sorbitol, thapsigargin (T9033), TMAO and taurine were from Sigma-Aldrich (Taufkirchen, Germany). Sodium 4-phenylbutyrate was from Axxora (Lörrach, Germany), ectoine from Biomol (Hamburg, Germany).

### Lentiviral Constructs and Production of Lentiviral Supernatants

**Figure 1:**
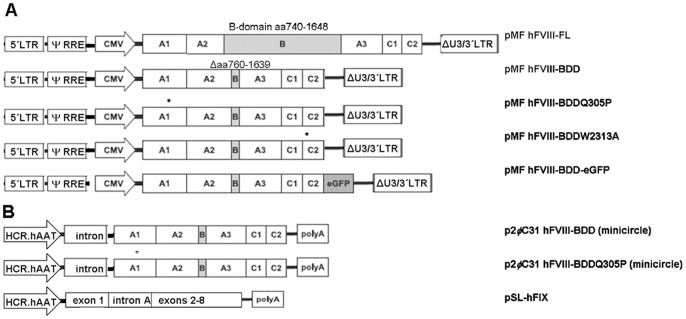
DNA constructs used in the *in vitro* or *in vivo* studies. Expression cassettes including domain structure of hFVIII-transgene or exon-structure of hFIX, promoter and enhancer elements. hFVIII-BDDeGFP has been previously described by Heinz et al. [25]. (A) *In vitro* lentiviral constructs were used for transduction of cell lines. (B) *In vivo* a hFIX-Minigene encoding plasmid was hydrodynamically injected in Hem B mice, whereas hFVIII-BDD or hFVIII-BDDQ305P expressing minicircles were produced for Hem A mouse studies. Asterisks indicate locations of point mutations.

cDNAs of human FVIII full length (FL) protein, FVIII B-domain deleted (BDD) protein [27], FVIII-BDD-eGFP [28] or a mutant FVIII-BDD protein were introduced in the lentiviral transfer vector pMF363 (Fig. 1). Vector production, concentration, and titration was performed as previously described [28]. Cells were kept for at least two weeks post transduction to ensure stable FVIII-expression prior to the start of the experiment.

### Generation of FVIII Mutants

Mutations were introduced by site directed mutagenesis (QuikChange® II XL Site-Directed Mutagenesis Kit, Agilent Technologies, Waldbronn, Germany) in lentiviral transfervector pMF363 [29] coding for FVIII-BDD. The resulting vectors, pMF363 FVIII-BDDQ305P and pMF363 FVIII-BDDW2313A, were used for transduction of HepG2 cells at a multiplicity of infection (MOI) of 10.

### Non Viral Vector Constructs

The human FIX mini-gene was used flanked by the hepatic locus control region 1, the human alpha-1 anti-trypsin promoter and a bovine growth hormone polyadenylation signal (HCR/hAAT-FIX) as described previously [26]. The expression cassette was introduced into a plasmid vector containing the pSL1180 backbone resulting in the pSL-FIX vector (Fig. 1).

Human FVIII-BDD or FVIII-BDDQ305P cDNA was excised from pMF363-vectors and cloned 3′ of a chimeric intron (from pCI-neo vector, Promega, WI) in a minicircle producer plasmid p2ΦC31 kindly provided by Mark Kay [30] (Fig. 1). Minicircles were produced as described [26], followed by a restriction digest (Plasmid-Safe DNase, Epicentre, WI) to eliminate contamination with residual plasmid backbone sequences.

### Chemical Chaperone Treatment of Heterologous Cell Lines

As expression systems we used CHO or HepG2 cells stably transduced with self-inactivating lentiviral vectors (MOI 10) carrying human FVIII-FL or –BDD, a mutant or enhanced Green Fluorescent Protein (eGFP)-tagged FVIII-BDD protein. Experiments were performed in 96 well plates with 1 to 2*10e4 cells per well. After cell adherence overnight media was supplemented with CC at different concentrations. After 72 h incubation with CC, levels of active FVIII were determined in the supernatants. To reveal toxic effects of CC, Hoechst 33342 staining (Thermo Fisher Scientific, Germany) was used to count adherent cells in 96 well plates. Experiments were performed in duplicates.

### FVIII and FIX Assays

FVIII activity in cell supernatant or mouse plasma was measured by the Coamatic chromogenic assay using normal human reference plasma for calibration according to the manufacturer (both Haemochrom Diagnostica, Germany). Human FVIII antigen in mouse plasma or cell lysates was quantified in comparison to recombinant human FVIII-BDD (ReFacto, Pfizer GmbH, Germany) using a sandwich ELISA (F8C-EIA FVIII:C, Affinity Biologicals, ON, Canada). 1 IU of ReFacto was defined as 100 ng FVIII Protein/ml. Human FIX concentrations were determined using an ELISA in which a monoclonal antibody to human FIX, clone HIX-1 (Sigma, St Louis, MO), was used as capture antibody. Peroxidase-conjugated polyclonal goat anti–human FIX (Affinity Biologicals, Hamilton, ON) was used as detection antibody. Mouse FIX levels were determined by a modified one-stage factor assay, by incubating 25 µl human FIX–deficient plasma with 25 µl automated activated partial thromboplastin time (aPTT) reagent (Dade Behring, Marburg, Germany) and a total of 25 µl of a test sample (diluted in human FIX-deficient plasma).

### Triton-soluble and Insoluble Fractionation of Intracellular FVIII

Intracellular FVIII was fractioned into Triton X-100-soluble and insoluble fractions as previously described [31] with minor modifications of the lysis buffers. CHO cells transduced with eGFP tagged hFVIII-BDD were treated with 50 or 100 mM betaine or control. After 72 h cells were washed twice in ice-cold PBS and lysed in ice-cold lysis buffer containing protease inhibitors [PBS/0.5% Triton X-100]. Cells were sedimented at 16,000×g for 30 min at 4°C. Supernatants were transferred into new tubes and saved as the detergent-soluble fraction. Pellets were washed twice with lysis buffer, were subsequently solubilized with SDS buffer (1% SDS instead of TX-100) and centrifuged for further 30 min. The supernatant was saved as Triton X-100 insoluble fraction. hFVIII distribution was analyzed by Western Blotting and indirect FVIII-ELISA (Affinity Biologicals, ON, Canada).

### Western Blot

Cell lysate fractions were centrifuged at 16,000×g for 20 min. Equal volumes were separated by SDS-PAGE (4–20% Gradient Gels, Thermo Fisher Scientific, Germany), transferred to nitrocellulose membranes and probed with anti-GFP rabbit serum (Roche Diagnostics GmbH, Mannheim Germany) or anti hFVIII Light Chain goat antibody (F-15). Internal control was done using anti-hGAPDH goat antibody (V-18, both SCBT Inc, Santa Cruz, CA).

### FACS Analysis

CHO cells expressing hFVIII-BDD-eGFP were incubated with 50 or 100 mM betaine for 48 or 72 h. Pellets were twice washed with PBS and resuspended in 1× CellFix (Becton Dickinson, Heidelberg, Germany). Intensity of hFVIII-BDD-eGFP expression (X mean GFP) in control versus betaine- or BHA-treated cells was determined in FACScan using CellQuest software (Becton Dickinson, Germany).

### Immunofluorescent Labeling and Microscopy

CHO cells expressing eGFP-tagged hFVIII-BDD proteins were seeded on coverslips in six-well plates and incubated for 72 h with or without betaine 100 mM. The eGFP signal of eGFP-FVIII fusion proteins could be directly detected by fluorescence microscopy. For immunofluorescent labeling of Golgi matrix protein 130 (GM130) cells were fixed with 3.5% paraformaldehyde, permeabilized with 0.1% Triton X-100 and blocked with 3% human serum albumin in PBS. Detection was performed by incubation with rabbit anti-GM130 antibody (H-65) and Alexa^543^-labeled anti-rabbit IgG secondary antibody (both antibodies, SCBT Inc., Santa Cruz, CA). Hoechst 33342 (Invitrogen) was used to stain the cell nucleus. Coverslips were mounted on to slides with Mowiol 4–88 (Carl Roth GmbH, Karlsruhe, Germany) and stored at 4°C. Images of immunostained cells were recorded on a fluorescence Axiovert 100 microscope (Zeiss AG, Jena, Germany).

### Statistical Analysis

Two-tailed paired Student’s t-tests or repeated measures ANOVA with Dunnetts Multiple comparison post test (GraphPad InStat Software 3.06, La Jolla, CA) were used for comparisons between CC-treated and untreated cells or mice. The Wilcoxon Signed Rank Test was employed for non-parametrical analysis.

## Results

### CC Supplementation Increases Secretion of Recombinant FL-, BDD- and eGFP Tagged BDD- FVIII

**Figure 2:**
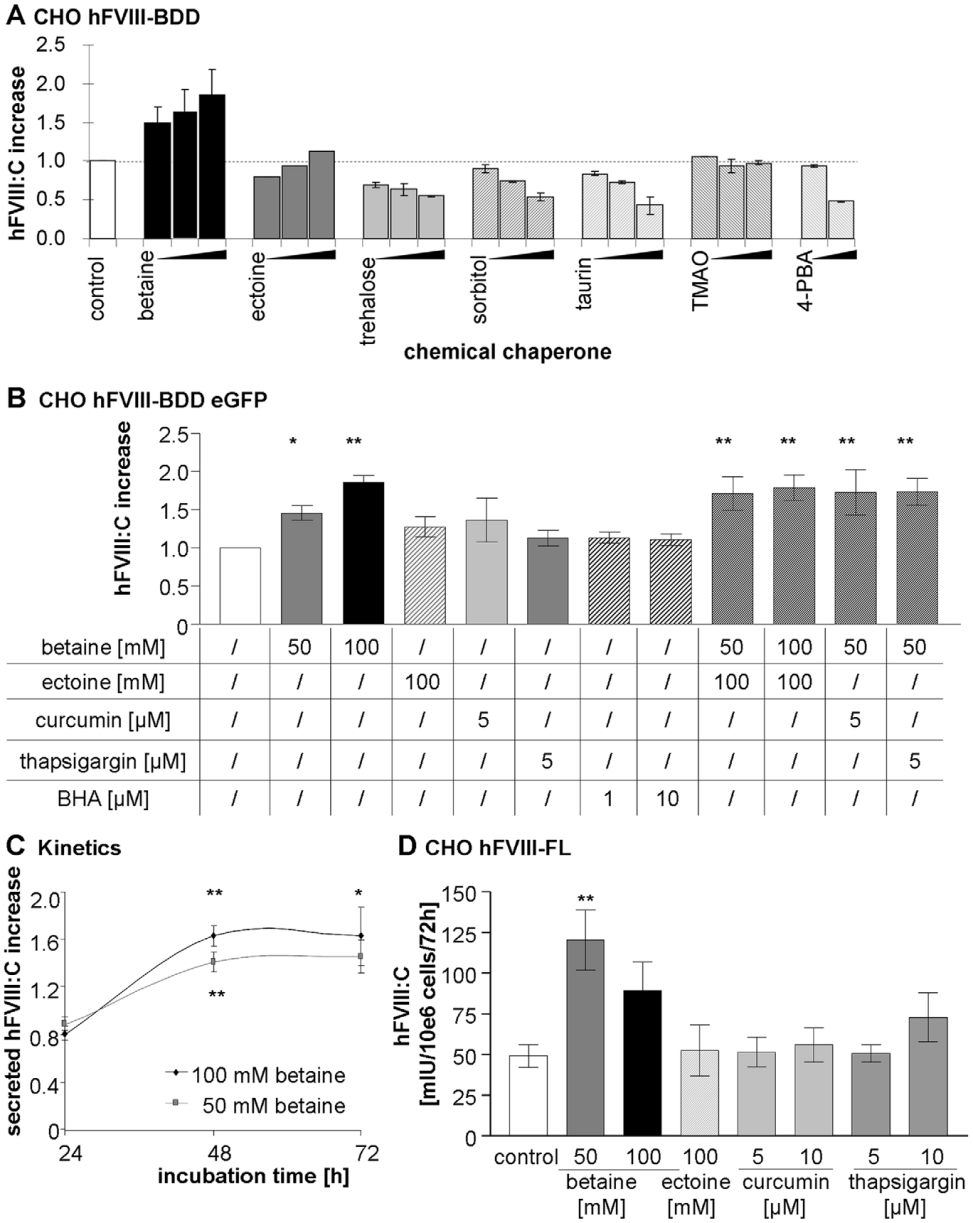
CC improve secretion of FVIII-BDD, FVIII-BDD-eGFP and FVIII-FL *in vitro*. Heterologous CHO cells were incubated with CC at different concentrations. FVIII activity was determined in cell supernatants after 72 h by chromogenic assay. (A) Effect of the following CC on human (h)FVIII-BDD secretion: Betaine (100; 50; 25 mM), ectoine (150; 100; 50 mM), trehalose (150; 100; 50 mM), sorbitol (150; 100; 50 mM), taurine (150; 100; 50 mM), trimethylamine N-oxide (TMAO;50; 25; 12,5 mM) and sodium 4-phenylbutyrate (4-PBA; 2; 0,4 mM). Number of experiments, n = 2. (B) Effect of betaine, ectoine, and the endoplasmatic ATPase inhibitors curcumin and thapsigargin on FVIII-.BDD-eGFP secretion. Butylated hydroxyanisole (BHA) is added as treatment control. n = 3. The mean FVIII secretion level ± SD of untreated hFVIII-BDD-eGFP expressing cells was 19±12 IU per 10e6 cells per 72 h. (C) FVIII-BDD-eGFP secretion into cell supernatants over time at different betaine concentrations. n = 3. (D) Influence of betaine, ectoine, curcumin and thapsigargin on FVIII-FL secretion 72 hours following drug supplementation. n = 3. All values are presented as means ± SEM. ANOVA test * *P*<.05; ** *P*<.001.

Initially, we tested members of different compound classes of CC for their effect on hFVIII-BDD expression in CHO cells: Polyols (sorbitol) [32], sugars (trehalose) [33], methylamines (betaine) [6] and trimethylamine N-oxide (TMAO) [4], free amino acids such as taurine [5] or amino acid derivatives (ectoine) [34] (Fig. 1A). Beside the former listed osmolytes, we tested the hydrophobic compound sodium-4-phenylbutyrate (PBA), which has already been tested as a chemical chaperone in a cystic fibrosis clinical trial [7]. Concentrations were selected based on reported data from conformational disorders, but were additionally pretested for cytotoxicity on CHO cells by determining cell count after 72 h of treatment using Hoechst 33342 nuclear stain (Sigma-Aldrich, Germany). Resulting test concentrations were: Betaine (100; 50; 25 mM); ectoine, trehalose, sorbitol and taurine (150; 100; 50 mM); trimethylamine N-oxide (TMAO; 50; 25; 12,5 mM) and sodium 4-phenylbutyrate (4-PBA; 2; 0,4 mM). The screening for secretion-supporting CC identified two promising candidates, betaine and ectoine. The other CC showed no effect on CHO cells (Fig. 2A).

In addition, another class of substances, the endoplasmic ATPase inhibitors, was also examined. These substances have been shown to successfully rescue trafficking of mutant CFTR *in vitro* [35] or to cause improved maturation of vasopressin V2 receptor mutants involved in nephrogenic diabetes insipidus [4]. We could show that addition of betaine, ectoine or curcumin all increased the secretion rate of eGFP-tagged hFVIII–BDD proteins in CHO cells (Fig. 2B). Thapsigargin alone showed no effect; a concentration of 10 µM was toxic to the cells. Addition of 100 mM betaine proved to be most effective, increasing the yield of secreted active hFVIII-BDD 1.9-fold. In contrast, the control substance butylated hydroxyanisol (BHA) had no effect on secreted FVIII yield (Fig. 2B). BHA is an antioxidant which was previously reported to improve cell integrity and survival when applied previously to induction of FVIII expression in a CHO cell line. It increased FVIII secretion by reduction of intracellular FVIII-accumulation and decreased oxidative stress evoked by induction. However, upon application to already FVIII producing cells no reduction of the apoptosis rate was detected [36]. As 100 mM betaine slightly inhibited cell growth in this experiment we supplemented betaine treatment at a concentration of 50 mM in combination with ectoine, thapsigargin or curcumin to achieve higher effects. CC combinations increased FVIII secretion synergistically, but did not exceed the effect of 100 mM betaine supplementation alone (Fig. 2B). Kinetics of 50 and 100 mM betaine treatment showed that the secretion rate increased significantly after 48 h hours of incubation (Fig. 2C). Betaine addition was also the most effective treatment in cells expressing FVIII-FL and led to up to 2.6-fold higher levels of secretion at 50 mM betaine concentration. 10 µM thapsigargin showed a modest effect on FVIII secretion, while curcumin showed no effect (Fig. 2D).

### Increased FVIII Levels are not Attributed to Improved Stability of FVIII in Cell Culture Supernatant or Plasma

**Figure 3:**
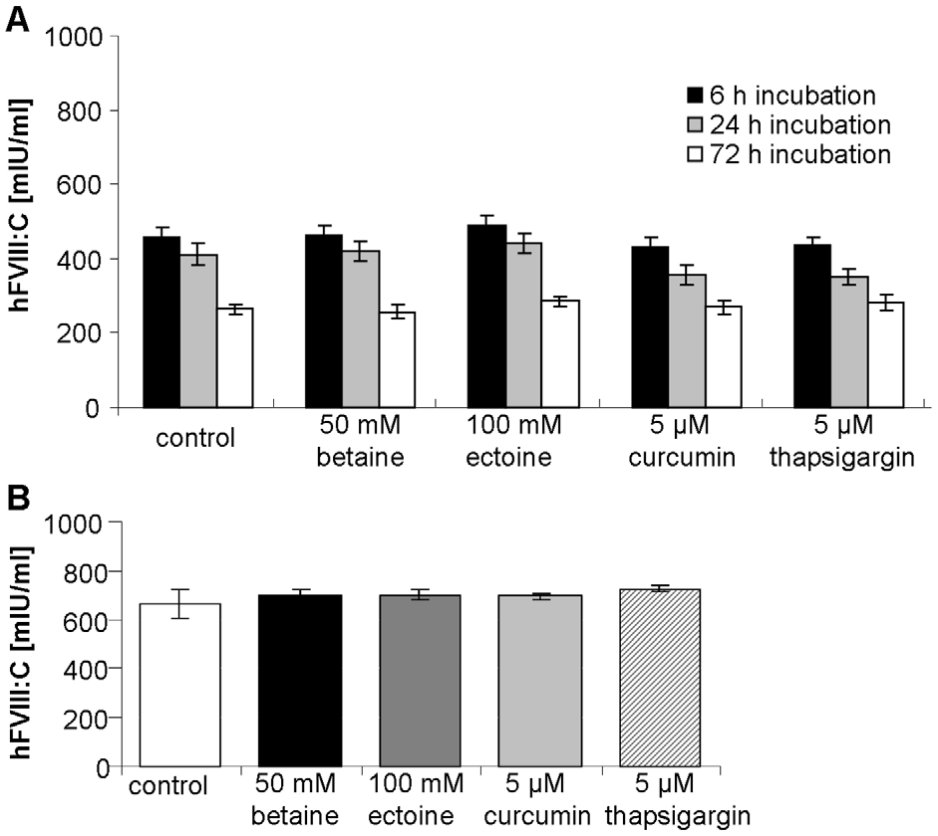
Influence of CC on FVIII stability in supernatant or plasma. CHO cells were incubated with supernatant from CHO cells expressing FVIII-BDD. Then representative CC concentrations were supplemented for 6, 24 h and 72 h (A) at 37°C. (B) Human normal plasma from Haemochrom Diagnostica (Germany) was incubated with CC for 72 h at 37°C. FVIII activity was determined using a two-stage chromogenic assay. Values are presented as means ± SEM, the number of independent experiments was n = 4.

To exclude the possibility that the cause of increased FVIII levels was a result of improved stability of extracellular FVIII, the supernatant of CHO FVIII-BDDeGFP cells was incubated with representative concentrations of betaine, ectoine, thapsigargin, and curcumin. FVIII activity was determined at different time points. Only ectoine, which is known as a protein stabilizer, showed a mild stabilizing effect on FVIII. There were no differences in FVIII-concentration after 6, 24 or 72 h following incubation with all other substances tested compared to non-treated controls (Fig. 3A). Incubation in normal human plasma (Haemochrom Diagnostica) did also not affect FVIII half life (Fig. 3B).

### Betaine Improves Intracellular FVIII Solubility and Corrects Processing of eGFP-tagged FVIII

**Figure 4:**
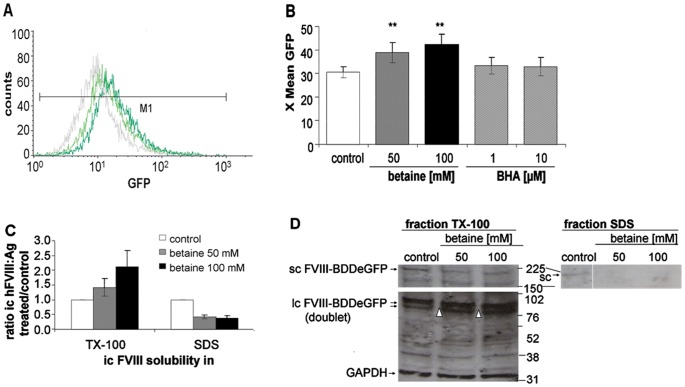
Betaine increases solubility of intracellular FVIII. CHO cells expressing eGFP-tagged FVIII-BDD protein were incubated with and without betaine. (A,B) Flow cytometry analysis was used to determine the eGFP-signal in untreated cells versus cells treated with betaine or control substance BHA. The mean eGFP intensity (X mean GFP) in the range M1 was used as distinctive parameter. (A) representative histogram after 48 h of treatment and (B) values after 72 h presented as means ± SEM of 3 independent experiments. ANOVA **P<.001. (C,D) After 72 h incubation cells were successively lysed in PBS/0.5% Triton X-100 and PBS/1% SDS. (C) FVIII antigen was determined in both fractions by indirect ELISA. (D) Triton X-100-soluble and insoluble fractions were separated on SDS-polyacrylamid gradient gels, and hFVIII light chains (lc), eGFP in hFVIII-single chain (sc) and GAPDH were detected by Western blot. Δ indicates lower band of hFVIII lc doublet.

FACS analysis of betaine treated eGFP-FVIII expressing cells revealed a shift in eGFP fluorescence intensity compared to control cells. Incubation with either 50 mM or 100 mM betaine increased expression of hFVIII-BDDeGFP after 48 (Fig. 4A) and 72 h (Fig. 4B), whereas butylated hydroxyanisole (BHA) treatment had no effect. We hypothesized that the betaine-induced higher secretion rate might correspond to higher intracellular concentrations, due in turn to increased intracellular FVIII solubility and higher levels of correctly folded proteins. To test this hypothesis, the amount of FVIII antigen in the detergent-soluble fraction (TX-100) and the detergent-insoluble fraction (SDS) of cell lysates was compared in ELISA and Western blot analysis. In CHO cells expressing an eGFP-tagged FVIII protein, 100 mM betaine treatment induced redistribution of intracellular FVIII antigen into the TX-100 soluble fraction (Fig. 4C). Less FVIII was found in the detergent-insoluble fraction, which is supposed to contain protein aggregates possibly targeted for degradation. During processing, FVIII is transported from the ER to the Golgi, where the FVIII single-chain is cut into a light and a heavy chain to form a secretion-competent heterodimer. To examine the effect of betaine on the maturity grade of FVIII we determined the relative amount of single chain versus light chain in both fractions by Western Blotting (Fig. 4D). These experiments revealed that in the detergent-soluble fraction the lower band of the light chain doublet (Δ) became stronger after betaine treatment, whereas the single chain band was weaker. In the SDS insoluble fraction only the single chain was detectable, but this also became weaker after betaine treatment.

### Betaine Facilitates ER to Golgi Transport of FVIII

**Figure 5:**
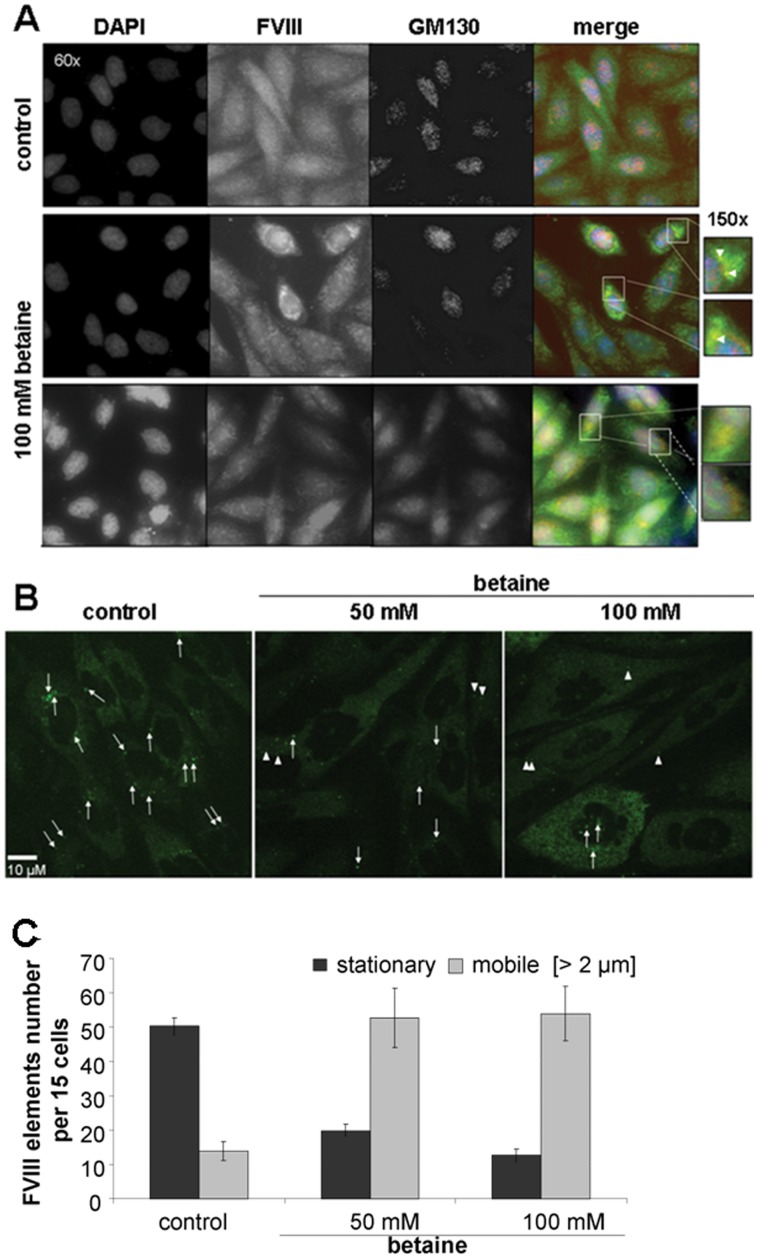
Betaine improves correct FVIII folding and ER-to-Golgi-Transport. CHO cells expressing eGFP-tagged FVIII-BDD protein were incubated with and without 100 mM betaine for 72 h. (A) Representative fluorescence microscopy analysis of intracellular FVIII distribution (original magnification x60). Yellow dots show co-localisation of FVIII (green) with golgi marker GM130 (red). Nuclear staining was done with Hoechst 33342 (blue). Two representative regions of colocalisation are magnified. (B and C) For life imaging cells were seeded on µ-slides VI (ibidi GmbH, Germany) prior to CC incubation. Cell analysis was provided in PBS imaging medium at 37°C by the Advanced Light Microscopy Facility of EMBL (Heidelberg, Germany) using PerkinElmer Improvision Ultraview Spinning disk confocal microscope combined with Hamamatsu CCD camera (original magnification x63, numerical aperture 1.4). Video sequences of 120 timepoints, one second each, were taken and analysed with Volocity Version 5.0.3 Build 4 (Improvision Ltd.). (B) Representative snapshots of Betaine and control conditions are shown. Arrows indicate all relatively stationary elements which were observed in the section, whereas arrowheads indicate only selected mobile FVIII elements, which were highly mobile. (C) Number of stationary and mobile (>2 µM distance) FVIII-eGFP elements were counted. The mean ± SD in 15 cells of three movies per condition is shown.

Immunofluorescence analysis revealed an altered intracellular distribution of FVIII proteins (Fig. 5A). In untreated cells the endoplasmic reticulum was distributed throughout the whole cytoplasm and the hFVIII-BDD-eGFP protein was relatively evenly distributed. In contrast, in betaine-treated cells the perinuclear localization of hFVIII-BDD eGFP was clearly more visible. Furthermore, in some cells, some FVIII accumulations could be seen to co-localize with the cis-Golgi marker GM130. Live cell imaging by spinning disk confocal microscopy of hFVIII-BDD eGFP also revealed that in the presence of betaine, there was a decrease in the number of static FVIII-positive structures, accompanied by an increase in the number of fast-moving punctuate elements (Fig. 5B and C).

### Betaine Increases hFVIII Plasma Levels in Gene-corrected FVIII Knockout Mice

**Figure 6:**
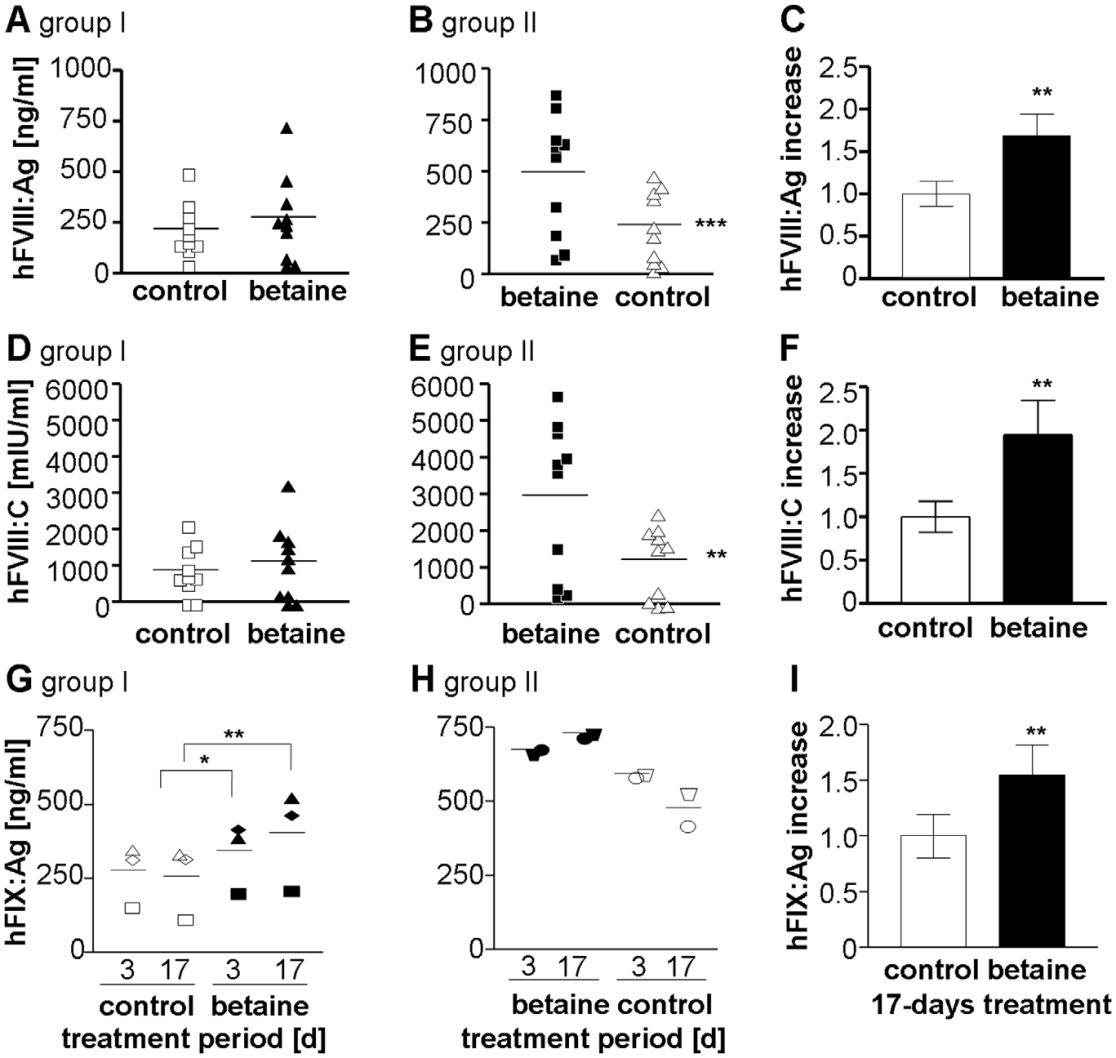
Betaine feeding improves hFVIII and hFIX secretion *in vivo*. (A–F) 24 hours post minicircle FVIII-BDD gene transfer the FVIII knockout mice received water without (group I) or with 2% betaine supplementation in the drinking water (group II, each n = 10). After 3 days plasma samples were collected and each group was monitored for human FVIII antigen (A and B) and related activity levels (D and E) and group treatment was switched. After another 3 days, plasma levels were tested again. (C and F) represent the calculated overall effect of betaine on FVIII antigen levels (C) or FVIII activity (D). square symbols indicate samples of the first measuring point, triangles the second one. (G–I) After reaching stable FIX expression levels following minicircle FIX gene transfer, FIX knockout mice were fed 2% Betaine-supplemented drinking water ad libitum in a crossover-study of two groups. After 3 and 17 days of treatment, retroorbitally collected plasma samples were monitored for human FIX antigen levels (G and H). (I) shows the overall change in FIX expression from both groups after 17 days of administration. All values are represented as mean ± SEM. Same symbols indicate samples of the same mouse at different time points; clear: tap water treatment (control), filled: betaine administration. Student’s t-test ((G) ANOVA). *P<.05, **P<.005, ***P<.0005.

To test the effect of betaine on FVIII secretion *in vivo*, we injected FVIII knockout mice with FVIII-BDD minicircle vectors. To better compare the course of hFVIII plasma levels under betaine and control conditions in the same mice, we decided for a crossover study design. We divided the mice into two groups, receiving either 2% betaine supplemented drinking water ad libitum or regular tap water as control. After 3 days, blood samples were retroorbitally collected and the treatment was switched between groups for further 3 days, ending with a second sampling. For analysis the parameters hFVIII:C, hFVIII:Ag, and mFIX:C in the murine plasma were measured. Following non-viral gene transfer, the highest expression levels are usually observed in the first 3 days of treatment [26] and then decrease until reaching stable expression levels between 4 and 8 weeks post treatment. Since human FVIII can induce an immune response in FVIII knockout mice, all experiments were performed within one week following gene transfer, to exclude the possibility of confounding effects of antibodies on FVIII expression levels. Despite the expected drop in FVIII over the time of the experiment, attributed to the gene transfer procedure, betaine supplementation stabilized and slightly increased the hFVIII antigen and hFVIII:C levels in group I mice (Fig. 6A and D). Group II showed significantly lower hFVIII levels after discontinuation of betaine treatment (Fig. 6B and E). Taken together, in 16 out of 20 mice, the FVIII:Ag levels were between 1.5 and 3.8-fold higher during the period of drug administration (Fig. 6C). The average increase in activity was 1.9-fold (Fig. 6F).

### Betaine Improves FIX Plasma Levels in Gene-corrected FIX Knockout Mice

A similar experiment was performed in FIX knockout mice following human FIX gene transfer. Since long-term expression of human FIX following minicircle gene transfer is not associated with an immune response, we started betaine supplementation when mice reached stable expression levels 8 weeks following FIX gene transfer. Additionally, using ELISA, we confirmed the absence of anti-FIX antibodies (data not shown). Betaine-supplemented, or regular drinking water, was provided for 17 days to equally-sized groups of mice. Betaine addition was then paused for one week, before re-starting each group on the alternative treatment. Human FIX ELISA revealed a 1.4-fold increase after 3 days with a further increase after 2 additional weeks of betaine treatment (Fig. 6G). After discontinuing betaine treatment, FIX levels dropped significantly (Fig. 6H). Overall, FIX levels increased on average 1.5-fold after 17 days of betaine treatment (Fig. 6I).

### Betaine Supplementation Rescues Secretion-defective FVIII Mutations *in vitro* and *in vivo*

**Figure 7:**
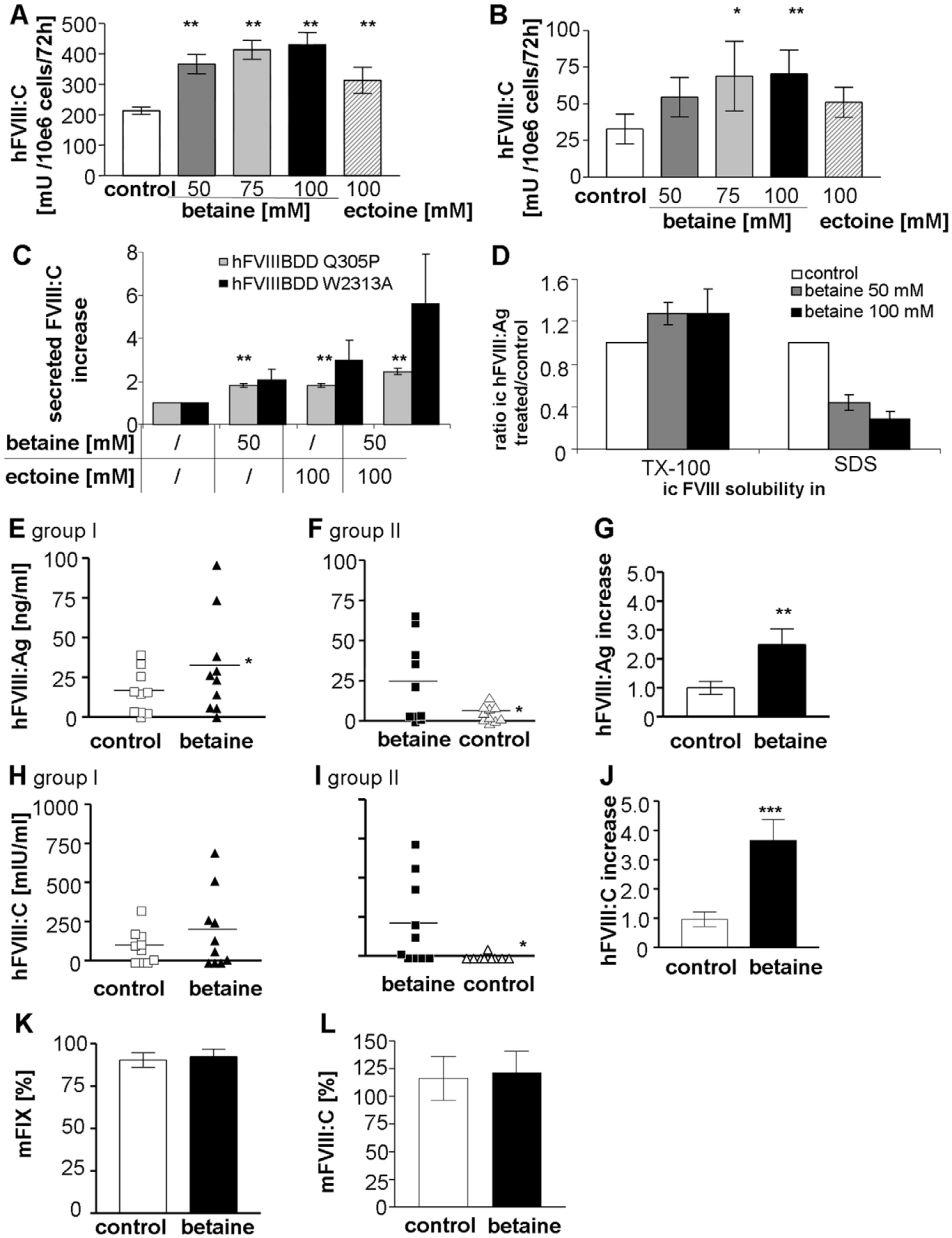
Rescue of mutant FVIII proteins *in vitro* and *in vivo*. (A–D) HepG2 cells expressing hFVIII muteins were incubated with CC for 72 h. Amount of hFVIII activity in cell supernatant of HepG2 cells expressing hFVIII-BDD Q305P (A and C) or hFVIII-BDD W2313A (B and C) was measured 72 h post betaine treatment. (A and B) show the supplementation of single CC and (C) betaine-ectoine combined treatment. (D) Post CC incubation HepG2 hFVIII-BDDQ305P cells were successively lysed in PBS/0.5% Triton X-100 and PBS/1% SDS. hFVIII antigen was determined in both fractions by indirect ELISA. (E–J) hFVIII-BDDQ305P injected Hem A mice were treated with 2% betaine ad libitum per os in a crossover-study of two groups (each n = 10). After 3 days of treatment hFVIII antigen and activity was measured and treatment was switched between mouse groups. 3 days later, plasma levels were tested again. (E and F) show hFVIII antigen levels, (H and I) the related hFVIII activity levels in plasma of group I or II. (G and J) represent the calculated overall effect of betaine on FVIII antigen levels (G) or FVIII activity (J). square symbols indicate samples of the first measuring point, triangles the second one; clear: tap water-administration (control), filled: 2% betaine administration. (K and L) Endogenous murine FIX levels in all injected FVIII knockout mice (K) and murine FVIII levels of all used FIX knockout mice (L) with and without betaine in the drinking water. Normal mouse levels were set to 100%. Values are presented as means ± SEM. (A–D) ANOVA; (E–H; K–L) Student’s t-test; (I–J) Wilcoxon signed rank test;*P<.05, **P<.005, ***P<.0005.

Since chemical chaperone treatment improved secretion of FVIII-BDD *in vitro* and *in vivo*, we asked whether betaine could also increase the secretion of secretion-defective FVIII proteins. Representatively, we chose two missense mutations that have been identified in hemophilia A patients and which are known to cause protein misfolding and ER retention. Amino acid substitution Q305P [37] is located in the A1 domain of FVIII, whereas W2313A [38] lies towards the C-terminus of the C2 domain (Fig. 7A and B). Mutant FVIII-BDD proteins were expressed in HepG2 cells. Cells were incubated with betaine or ectoine for 72 h and FVIII activity was determined in a two stage assay. The levels of both mutant FVIII proteins in the supernatant increased up to 2-fold with increasing betaine concentrations (Fig. 7A and B). Supplementation of ectoine also resulted in 1.5-fold higher levels. In this assay, 100 mM betaine inhibited cell proliferation. Therefore we combined 50 mM betaine and 100 mM ectoine treatment. Both had a slightly synergistic effect in HepG2 FVIII-BDDQ305P-expressing cells (2.5-fold) as well as in HepG2 FVIII-BDDW2313A-producing cells, with minimal cell growth inhibition (Fig. 7C). The TX-100/SDS solubility assay of HepG2 FVIII-BDDQ305P showed that more than 95% of the mutant hFVIII-antigen in the lysates was found in the TX-100 insoluble fraction (data not shown). Betaine induced a redistribution of intracellular FVIII antigen into the TX-100 soluble fraction similar to the effect observed in hFVIII-BDD secreting CHO cells, but to a lesser extent (Fig. 7D). 100 mM betaine treatment reduced the amount of hFVIII-antigen trapped in the detergent-insoluble fraction about 60 percent.

*In vivo* we examined betaine treatment in FVIII knockout mice expressing mutant human FVIII-BDDQ305P. The experimental design was identical to the human FVIII studies in mice described above. The starting plasma levels of FVIII-BDDQ305P were around 10-fold lower compared to FVIII without the mutation (Fig. 6). In group I the mice which switched from pure tap water to betaine supplementation for 3 days showed an increase in FVIII-BDDQ305P activity and antigen levels. In the mice treated with betaine in the first part of the study we could only detect FVIII activity in a single mouse after switching to pure tap water for 3 days. Therefore only animals which received betaine in the end of the experiment retained residual FVIII activity 8 days post injection (Fig. 7H and I), which corresponded to higher FVIII antigen levels (Fig. 7E and F).

### Betaine has no Influence on Endogenous Mouse Coagulation Factors VIII or IX

To examine betaine influence on endogenous plasma proteins in the mouse models, we additionally monitored mouse coagulation factors VIII or IX in all collected samples from all treatment groups. Betaine treatment did not change murine FVIII or FIX activities (Fig. 7K and L).

## Discussion

Here we show that supplementation with CC can improve FVIII secretion both in cell culture and *in vivo*. In initial screening of CC candidates, the substance betaine showed the highest potency, in that it doubled the amount of secreted FVIII. Secreted FVIII was fully functional, which indicates no major limitation in other posttranslational processes required by the secreted protein. Other substances known to improve protein secretion either had no effect, or only a very mild effect, on secretion of FVIII. One possible explanation for this finding is the significant cellular toxicity observed with CC concentrations needed to enhance FVIII trafficking for these substances. On the other hand, experiments exploiting other CCs are derived from secretion studies of mis-folded mutant proteins. Here, we tested a system in which FVIII is already trapped in the ER as a result of an overload of the cell’s folding capacity, in turn due to high levels of recombinant protein over-expression. Betaine therefore appears to be an attractive option to overcome current limitations in pharmaceutical FVIII-production. In fact, a previous report described that betaine preserves cell viability at hyperosmolar conditions and therefore adds to increased productivity of thrombopoietin in CHO cells under these conditions [39]. In addition, we observed that ectoine, thapsigargin and curcumin were able to further increase FVIII secretion when applied in combination with betaine. While ectoine is an osmolyte similar to betaine, the ER-ATPase inhibitors thapsigargin and curcumin reduce calcium levels in ER, which in turn alters interactions with calcium-dependent chaperones such calnexin and calreticulin [35], or affects the structure of MCFD2. Both of these scenarios might therefore produce additive effects.

In previous studies we observed that BDD FVIII concentrations in the ER are higher, and FVIII transport is more likely to occur by bulk flow, compared to FVIII FL [13], [28]. By contrast, the transport of the FL protein is primarily receptor mediated using LMAN1 and MCFD2 [40]. Different optimal betaine concentrations in FVIII-FL and –BDD expressing cell lines might therefore result in differences in protein trafficking. Supportingly as the administration of thapsigargin only improved FVIII-FL secretion, it seems to be more susceptive for alteration of the calcium level in the ER than FVIII-BDD. The B-domain contains most of the N-glycans which had to pass the quality control by the calnexin/calreticulin cycle. Both endoplasmic lectins are also known for prolonged binding of apparently misfolded glycoproteins. Possibly thapsigargin could influence these calcium-sensitive chaperone interactions with the B-domain and release FVIII-FL. An alternative hypothesis to consider could be differential betaine sensitivity of cell clones.

Betaine is proposed to stabilize proteins according to the “preferential exclusion“ model [3], [41]. The model hypothesizes that compatible solutes are excluded from the protein hydrate-sheath. The osmolytes are repelled form the peptide backbone due to osmophobic interactions, whereby water molecules are pushed to the protein surface, resulting in the protein adopting a more compact conformation [42]. This process reduces inter-protein interactions by surface-exposed hydrophobic patches, leading to aggregate formation and could probably alter chaperone accessibility to proteins. In our study, betaine increases the proportion of correctly folded and processed FVIII in cell lysates. The total amount of detergent-soluble intracellular FVIII was higher, whereas the amount of FVIII in the insoluble fraction decreased. The improved solubility possibly points to an augmentation of transport-competent FVIII in the ER and a reduction of protein aggregation. Western blot analysis revealed that betaine also improved the maturity of FVIII, as more single-chain was cut into light and heavy chains, a process which requires transport from the ER to the Golgi. Likewise Santana et al. [8] observed that betaine showed best results amongst various other CC in reversing the solubility and restoring the enzymatic activity of the mutant alanin:glyoxylate-aminotransferase protein (AGXT*LTM). Misfolded AGXT*LTM tends to build inactive aggregates in primary hyperoxaluria type 1 disease. The enzyme deficiency results in toxic oxalate accumulation in the kidney of homozygous patients. The authors could demonstrate that betaine induced an increase of soluble AGXT*LTM in COS-7 cell lysates accompanied by a reduction of insoluble protein. However the total protein amount remained unaffected. A similar effect was also shown for another methylamine, TMAO, in the correction of myocilin-causing glaucoma [43]. In transfected cells TMAO improved the secretion of the aggregation-prone D384N mutant through increased solubility. Additionally, TMAO induced redistribution of the mutant protein from an ER-enriched to Golgi-enriched fraction in a density gradient system.

Our immunofluorescence staining suggested that the major portion of the translated FVIII protein is retained in the ER and does not reach the Golgi apparatus. These results are in accordance with our previous work in COS-7 cell lines [13]. If FVIII passes ER quality control, it is packaged into COPII-coated carriers, mediated by LMAN1/MCFD2-receptors [39], [44], prior to these carriers budding from the ER. These vesicles are transported to the ERGIC and further on to the Golgi apparatus [45]. We observed in this study that betaine treatment partially changes the distribution of FVIII throughout the cell towards an increased perinuclear staining with punctate FVIII structures co-localizing with the cis-Golgi marker GM130. GM130 itself is known to shuttle between the ERGIC and Golgi [46], therefore one possibility is that the punctate structures of co-localization could be FVIII-vesicles in transit between the ERGIC and cis-Golgi. Live imaging analysis supported these findings, as betaine treatment led to a reduction in the number of FVIII-positive stationary elements, presumably representing accumulations of FVIII in ER exit sites or ER-retained FVIII aggregates, with the concomitant increase in active transport of FVIII.

Following these promising results in cell culture we wanted to explore whether betaine treatment could also increase protein secretion *in vivo*. Betaine is a natural methyl group donor in homocysteine- and methionine-metabolism. The physiological source of betaine is food (sugar beets, wheat germ, shrimps, spinach, etc.) [47], or it is generated by oxidizing choline [48]. It has approval as a dietary supplement in the USA and is commonly used for commercial feeding. Furthermore, betaine is easily administered by oral application and is already routinely used in patients suffering from hyperhomocysteinemia [48]. Since 2001 betaine has been classified as orphan drug for treatment of homocystinuria by the EMA. Additional to its function as a methyl donor, betaine is also being evaluated for its function as a CC in a phase II study in patients with primary hyperoxaluria type 1 (ClinicalTrials.gov Identifier NCT00283387). The disease is caused by a point mutation, which leads to a secretion defect of the liver enzyme alanine-glyoxylate aminotransferase. Betaine has also been tested in pre-clinical settings in cystic fibrosis [6]. Several mutations leading to a trafficking defect are also known for FVIII [37], [38]. Although these described mutations are rare, 78% (132 out of 170 with documented antigen levels) of missense mutations listed in the hemophilia A database (Dr. Geoffrey Kemball-Cook, The Haemophilia A Mutation, Structure, Test and Resource Site, http://hadb.org.uk, accessed August 18, 2011) have reduced antigen levels of below 50%, possibly as a consequence of impaired protein secretion. For this reason, a treatment strategy to improve protein trafficking could potentially benefit a wider patient population. Whether the improved processing and secretion of mutant FVIII protein might increase the risk for inhibitor formation has not been investigated at this point. In patients with missense mutations protein secretion is impaired due to intracellular retention of the mutant FVIII, but there is still residual protein in circulation [49]. Therefore CC-rescued protein would not be foreign for the immune system. In theory, neo-antigens could occur due to release of protein that otherwise would never have reached the circulation. Although we cannot provide data addressing this concern, we believe that this risk is negligible since the effect of CC is relatively mild and aims on supporting correct protein folding rather than release of not correctly processed FVIII. Here, over-expression in single cells as observed in gene therapy might carry an even higher risk.

In this study we investigated two representative mutations, one located in the A1 domain (Q305P) and the other in the C2 domain (W2313A) of FVIII. Q305P lies within the binding site for immunoglobulin-binding-protein BiP (T291-F309) and disrupts the interaction with this chaperone [15], [37]. hFVIII-BDDW2313A is rapidly degraded intracellularly but has 90–100% of the specific activity of wild-type FVIII-BDD [38]. For both mutations, betaine supplementation increased secretion of functional protein in cell culture. Q305P was further evaluated in FVIII deficient mice. For these experiments, human mutant coagulation factors were expressed in FVIII deficient mice using non-viral gene transfer. Addition of betaine to the drinking water resulted in higher FVIIIQ305P levels, but when betaine treatment was discontinued FVIII activity returned to baseline.

Similarly, betaine treatment also increased FVIII levels in mice expressing the human FVIII-BDD without missense mutation. In contrast, murine FVIII levels remained unchanged. We hypothesized that endogenous murine coagulation factors would be less affected by betaine treatment. Physiological expression might be more closely regulated and impaired folding as a result of ER capacity overload seems unlikely compared to hFVIII over-expression in limited numbers of liver cells in the gene transfer setting. To explore this difference, we used a similar gene transfer approach with the human coagulation FIX for non-viral gene transfer. Betaine increased recombinant FIX levels but did not influence endogenous murine FVIII. Although FIX is less affected by limitations of the ER to Golgi trafficking system, heterologous overexpression could also lead to impaired folding and processing of FIX in the ER.

Similar to FVIII, FIX mutations with impaired intracellular protein processing have also been described [50]. The same is true for mutations affecting von Willebrand factor [51] and combined FV/FVIII deficiencies, where an LMAN1/MCFD2 defect causes FVIII retention in the ER [44]. All of these might therefore be targets for CC-improved protein secretion.
